# Cochlear Implants and the Aided Audiogram: A Retrospective Study Comparing Performance Across Device Manufacturers

**DOI:** 10.3390/audiolres15040079

**Published:** 2025-07-02

**Authors:** Nicole Hope Capach, Noam Zigdon, Taylor A. Payne, Jonathan D. Neukam, Yeonjoo Choi, Hong Ju Park, William H. Shapiro, Mario A. Svirsky

**Affiliations:** 1 Department of Otolaryngology Head and Neck Surgery, New York University Grossman School of Medicine, New York, NY 10016, USAmario.svirsky@nyulangone.org (M.A.S.); 2 Department of Hearing and Speech Sciences, Vanderbilt University Medical Center, Nashville, TN 37232, USA; 3 Department of Otorhinolaryngology-Head and Neck Surgery, Asan Medical Center, University of Ulsan College of Medicine, Seoul 05505, Republic of Korea

**Keywords:** cochlear implants, aided audiograms, T-levels, electrical stimulation levels, cochlear implant programming, audiological evaluations, hearing outcomes

## Abstract

**Background/Objectives**: We investigated: (1) differences in CI-aided thresholds and speech perception scores among cochlear implant manufacturers and (2) the relationship between CI-aided thresholds and speech perception. **Methods**: We analyzed exploratory data from NYU and a confirmatory data set of 120 CI-aided audiograms from the ASAN clinic. CI-aided soundfield evaluations were compared between manufacturers (Cochlear, Advanced Bionics, MED-EL) using 5- and 6-pure-tone average thresholds; percentage of patients with average thresholds above 35 dB HL; speech perception scores; and correlations between thresholds and speech perception. **Results**: Compared to Cochlear users, MED-EL and Advanced Bionics users had significantly higher (poorer) pure-tone averages (26.7 dB HL for Cochlear vs. 30.0 dB HL for AB and 34.6 dB HL for MED-EL at NYU; 29.0 dB HL for Cochlear vs. 36.5 dB HL for MED-EL at ASAN), and higher incidence of 5- or 6-PTAs above 35 dB HL (1.6% vs. 23.4%/47.1% at NYU; 11.2% vs. 60.0% at ASAN). Word and sentence scores were significantly higher for the Cochlear group when compared to the MED-EL group. Speech scores were higher for manufacturers that recommend the use of behaviorally-measured T-levels (Cochlear) rather than estimated T-levels (AB and MED-EL). Significant negative correlations existed between CI-aided thresholds and speech scores. **Conclusions**: Significant differences in CI-aided thresholds and speech perception were observed between manufacturers, potentially related to brand-specific T-level programming approaches.

## 1. Introduction

A cochlear implant is a neuroprosthetic that bypasses a damaged cochlea by using electrical pulses to stimulate the auditory nerve, thereby restoring hearing sensation. Briefly, acoustic sounds are picked up by an external mic, digitally processed, and then mapped to corresponding electrical stimulation levels. Arguably, one of the most important clinical procedures in CI programming is the perceptual determination of electrical stimulation levels [1], which is completed by specifying a lower electrical stimulation level (T or THR Level) and an upper electrical stimulation level (C, M, or MCL level). For simplicity, lower electrical stimulation levels, also known as programmed electrical thresholds, will be referred to as T-levels within this paper.

The CI-aided soundfield audiogram is a clinical tool that is commonly used to verify audibility among the CI patient population during an audiological evaluation. This aids in the confirmation of CI programming. Specifically, CI-aided soundfield thresholds give insight into patients’ access to soft sounds and therefore can verify the accuracy of lower electrical stimulation levels [2]. This is because, when T-levels are behaviorally measured, CI-aided thresholds are predictable within a small range, based on programming settings (such as sensitivity or T-SPL, the programmable setting that specifies the sound pressure level associated with T-level stimulation), the acoustic-to-electric transform function and accuracy of T-level measurements. For each stimulated electrode, softer acoustic sounds are mapped closer to the lower electrical stimulation level (T-level) and louder acoustic sounds are mapped closer to the upper electrical stimulation level. Speech processors are designed to map a predetermined sound level, such as 25 dB SPL for Cochlear devices, to the recipient’s T-level stimulation [2,3,4]. In other words, the speech processor will provide T-level stimulation when the corresponding acoustic input reaches the predetermined level (e.g., 25 dB SPL), regardless of anything else. This is a mechanistic fact about the performance of an electronic device that has nothing to do with human physiology, perception, or behavior. Thus, the acoustic input level that results in T-level stimulation is unaffected by factors such as electrode array positioning (perimodiolar, mid-scala, or lateral wall), the presence of bone growth, otosclerosis (or other middle or inner ear pathologies), the etiology of hearing loss (congenital or acquired), or the duration of profound deafness prior to implant activation. If the acoustic input reaches a predetermined level, the electrical output will be at the threshold level (T-level). The consistency in CI-aided thresholds is maintained regardless of the absolute value of the electrical threshold, be it 1 microampere or 1 milliampere, or whether it is 5 or 250 clinical units. To better understand this, it is helpful to visualize an example of the acoustic-to-electric transform function.

**Figure 1:**
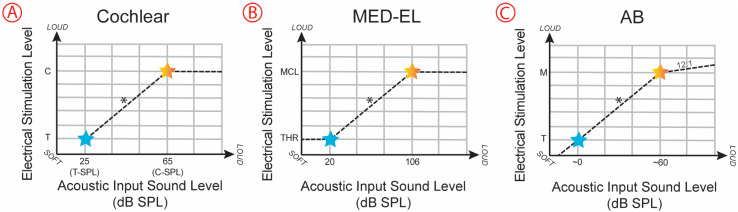
Acoustic-to-electric transform functions for the three cochlear implant manufacturers when default settings are used. Acoustic input sound (on the x axis) is mapped to electrical stimulation levels (on the y axis). Threshold level stimulation is denoted by a blue star and upper stimulation level is denoted by an orange star. * The functions may vary due to both programmable settings and manufacturer-specific non-programmable settings, such as TSPL, CSPL, IDR, gain, sensitivity, map law/loudness growth, and automatic gain control. Panel (**A**) illustrates that for Cochlear devices, T-level is mapped to T-SPL. Anything below T-SPL (softer than 25 dB SPL in this default example) does not result in stimulation. Panel (**B**) illustrates that for MED-EL devices, THR (T-level) is mapped to 20 dB SPL. THR is the minimum stimulation level, and the MED-EL device provides continuous stimulation at this level. Panel (**C**) illustrates that for AB devices, T-level is mapped to about 0 dB SPL (~60 dB SPL minus the acoustic input dynamic range (IDR) default of 60 dB). Stimulation continues below T-level for sounds that are softer than 0 dB SPL, assuming T-level is not set to 0 µA.

Figure 1 depicts a simplified presentation of acoustic-to-electric transform functions, adapted from Vaerenberg, Govaerts, Stainsby, Nopp, Gault and Gnansia [4]. For each electrode, a fixed acoustic input sound level is mapped to the lower electrical stimulation level (T-level) by the speech processor. In reality, the function can be much more complicated, and specifics of how acoustic input is mapped to electrical stimulation vary by manufacturer and depend on additional programmable settings. Even so, due to the formulaic approach of the acoustic-to-electric transform functions, when T-levels are behaviorally measured, CI-aided soundfield thresholds will cluster within a small range, based on programming settings of the device. In other words, programmed T-levels have a direct, mechanistic impact on CI-aided audiograms.

There are two broad approaches to determining T-levels: measurement and estimation. Behavioral measurement of clinical T-levels does not involve presentation of sound, but rather, T-level is determined through presentation of electric stimulation on a single electrode. T-levels can be behaviorally measured for all electrodes or for a subset of electrodes across the array using techniques similar to threshold audiometry [5] or using a counted T approach [6]. If using a subset of electrodes, any remaining electrodes are then interpolated [5,7,8]. Within this paper, we use the term “estimated T-levels” to refer to any programmed T-levels that did not result from direct measurement of T-levels (either in the electrode itself or in neighboring ones). A notable advantage of using measured T-levels, rather than estimated T-levels, is that direct measurement of variables inherently provides greater accuracy and reliability than estimation-based approaches, reducing uncertainty and systematic error. On the other hand, even though estimation may be less accurate than measurement, it can also be faster. This is because estimation of T-levels is typically performed as a percentage of the upper stimulation level (C, M. or MCL level), and calculation of those percentages is performed instantaneously by the fitting software. Although interpolation may be used for some electrodes when a patient has measured-T levels, it is unlikely to result in systematic biases (over or under-estimation of T-levels), whereas estimating T-levels at a percentage of upper stimulation level (or keeping them at minimum) may result in such biases, or in errors greater than those that may result from interpolation. For more information about how T-levels are programmed, please refer to Section 2.2 Clinical Fitting Procedures.

The approach to T-level programming varies between manufacturers, which may result in manufacturer differences in the CI-aided audiogram. When T-levels are estimated, rather than behaviorally measured, there is no assurance that CI-aided thresholds will be clustered around a specific value. This study aims to investigate potential manufacturer differences in CI-aided audiograms and the extent to which these differences may affect CI-aided speech perception.

## 2. Materials and Methods

### 2.1. NYU Exploratory Data Set

An initial exploratory study was conducted using data from our clinic at New York University Cochlear Implant Center (NYU). Cochlear implant surgeries completed at NYU between 1 January 2014 and 28 May 2021 were compiled. Implant recipients less than 18 years of age and SSD patients were excluded, as the vast majority of these patients do not have CNC (Consonant-Nucleus-Consonant) or AzBio scores (other speech materials were used). All patients included in this paper were implanted post-lingually as adults. Patients with NF2 or a vestibular schwannoma were excluded as these conditions represent atypical cochlear implant cases where nerve health is more likely to be compromised [9] and speech perception scores, as a group, are poorer than those of the general cochlear implant clinical population [10,11]. This study was approved by the NYU Institutional Review Board.

For the identified patients, we collected and tabulated CI-aided audiometric thresholds and CI-aided speech testing data (CNC words in quiet, AzBIO sentences in quiet, and AzBIO sentences in noise) for the first 2 CI-aided audiological evaluations post-implantation. Formally, these time points are the 3-month and 12+ month evaluations. Audiological evaluations followed the NYU standard clinic protocol. All evaluations were completed in soundproof booths. CI-aided thresholds were measured in free-field using warble tones from 250 Hz to 6000 Hz. The Hughson-Westlake procedure was used [12] with an ascending step size of 5 dB and a descending step size of 10 dB. According to the NYU protocol, ear-specific results were obtained using recorded speech material presented at 60 dB A with a +10 SNR for the noise condition. For patients with bilateral CIs and no residual hearing, the contralateral CI processor was removed. For patients with residual hearing, earplugs (or earplugs together with earmuffs) were used to appropriately attenuate the speech stimuli.

One hundred and seventy-two patients were included, resulting in a total of 316 CI-aided evaluations for 186 ears. Thirty patients were unilaterally implanted, 50 patients were bilateral, 81 patients were bimodal, and 7 patients had modalities that changed over the first year of device use (1 was unilateral to bimodal, 2 were unilateral to bilateral, 1 was bimodal to unilateral, and 3 were bimodal to bilateral). Of the bilateral CI users, 16 patients had both ears included in this dataset. Out of the 184 total ears, 174 had an early evaluation (formally known as the 3-month evaluation) and 142 had a late evaluation (formally known as the 12+ month evaluation). The average age at implantation was 60.7 years (range 20.3 to 93.5 years). In practice, these timepoints span a range: on average, 3-month evaluations were completed at 3.96 months (range 2.93 to 7.46 months) after CI surgery and 12+ month evaluations were completed at 15.58 months (range 8.44 to 54.24 months) after CI surgery. Note that CI surgery is about 1 month before the initial stimulation of the device.

One hundred and four patients (109 ears and 188 CI-aided evaluations) had a Cochlear device, forty-six patients (54 ears and 94 CI-aided evaluations) had an AB device, and nineteen patients (20 ears and 34 CI-aided evaluations) had a MED-EL device. Audiological evaluations were separated into groups by device manufacturer. Both the age of implantation and early and late evaluation timepoints were similar across the groups. The average age at implantation was similar across groups: 60.1 years (range 21.7 to 93.0 years) for the Cochlear group, 62.1 years (range 20.3 to 93.5 years) for the AB group, and 53.2 years (range 24.1 to 75.7 years) for the MED-EL group. The average time of the early (3 month) evaluation was 3.9 months (range 2.9 to 6.1 months) after CI surgery for the Cochlear group, 4.0 months (range 3.2 to 7.5 months) after CI surgery for the AB group, and 3.8 months (range 3.4 to 6.6 months) after CI surgery for the MED-EL group. The average time of the late (12+ month) evaluation was 14.8 months (range 8.4 to 54.2 months) after CI surgery for the Cochlear group, 15.7 months (range 8.7 to 46.7 months) after CI surgery for the AB group, and 19.2 months (range 12.6 to 50.8 months) after CI surgery for the MED-EL group.

### 2.2. ASAN Confirmatory Data Set

Based on the results from the exploratory study, we conducted a confirmatory study after pre-registering our hypotheses and analysis methods (https://aspredicted.org/44S_MYW, created 18 July 2023). The confirmatory data set was provided by the Otology Clinic of Asan Medical Center in South Korea (ASAN) on 8 August 2023. This data was previously collected by the ASAN clinic [13]. Our investigators did not have access to the data prior to preregistration. This data set includes 120 adult CI patients who had CI surgery between August 2000 and January 2016. Each patient has 1 datapoint at the 2-year timepoint. Ninety-eight patients had a Cochlear device, 20 patients had a MED-EL device, and 2 patients had an AB device. Because there were only 2 AB patients in this dataset, they were not included in the analysis. Nearly all patients (N = 119) were unilaterally implanted, with 1 patient being bilaterally implanted. In the case of the bilaterally implanted patient, only the first CI was included in this data set. All patients were experienced CI users with the average age at implantation at 51.3 ± 13.2 years (range: 21.0–80.3 years). Patients were tested unilaterally in a soundfield within a soundproof booth. For patients with residual hearing, ear-specific results were obtained by using earplugs, earplugs + earmuffs, or masking for patients with residual hearing. CI-aided thresholds were measured using warble tones from 250 Hz to 4000 Hz. Open-set monosyllabic words and high-predictability sentences were tested in quiet using monitored live voice presented at speech reception threshold (SRT) +40 dB.

Analysis of the ASAN data set builds upon the exploratory findings of the NYU data set through a confirmatory, pre-registered research design. While analysis of the NYU data yielded promising results, those findings were obtained through exploratory analyses that were not specified in advance. Such exploratory research, though valuable for generating hypotheses, is susceptible to various forms of bias, including p-hacking, HARKing (Hypothesizing After Results are Known), and selective reporting of outcomes [14]. To the extent that a pre-registered analysis confirms the exploratory findings, our confidence in the latter is reinforced for several key reasons. First, by publicly documenting our hypotheses and analytical approach before having seen the ASAN data, we eliminated researchers’ degrees of freedom that can inflate false positive rates [15]. Second, our commitment to report all pre-specified analyses regardless of outcome mitigates publication bias concerns. Third, the replication of our key findings under these more stringent methodological constraints demonstrates that the observed effects are robust and not artifacts of analytical flexibility [16].

### 2.3. Clinical Fitting Procedures

NYU protocol specifies the following clinical mapping appointments: activation day 1, activation day 2, 1 week, 1 month, 2 months, 6 months, 12 months, and then annually. Additional mapping appointments are made on an as-needed basis. NYU CI-aided evaluations are completed at 3 months and 12 months. ASAN protocol specifies the following clinical mapping appointments: initial activation, 3 months, 6 months, 12 months, and then annually. ASAN CI-aided evaluations are completed at 2 years.

Each cochlear implant manufacturer has specific programming approaches that often vary both within and across clinics. For example, there are distinct instructional differences when setting the upper stimulation level (C, M or MCL): For Cochlear devices, C levels is set at “loud but comfortable”; For MED-EL devices, MCL levels is the “maximum comfort level”; For AB devices, M levels is set at “most comfortable level” [2,17]. There are also different guidelines for setting the lower stimulation levels (T or THR): For Cochlear devices, T-level is set at the lowest level with 100% detection [2,18]; For MED-EL devices, THR level is typically estimated as a fixed percentage of MCL, with 0%, 8%, and (more recently) 10% being the most common values [2,18,19]; For AB devices, T-level is typically estimated at 10% of M level [2,17,18]. Consistent with both manufacturer and NYU/ASAN clinic fitting procedures, all patients with Cochlear devices had behaviorally measured T-levels, and nearly all patients with MED-EL or AB devices had estimated T-levels (with 5 exceptions in the NYU data set where T-levels were measured for MED-EL or AB patients).

Within the Cochlear group, the approach for finding T-levels varies (i.e., threshold or counted T approach). Additionally, in some patients, only a subset of electrodes may be measured, and the remaining electrodes are interpolated from the measured values. The latter applies to the upper stimulation levels as well (for all patients, whether using Cochlear, AB, or MED-EL devices). Within the NYU and the ASAN clinics, behavioral measures are used for setting upper stimulation levels (C, M or MCL level) and may include loudness scaling individual electrodes, loudness scaling of groups of electrodes (AB), loudness scaling in “live listen” mode, and loudness balancing across electrodes. To our knowledge, no patients within this study were programmed using electrically evoked stapedial reflex thresholds (eSRTs) or electrically evoked compound action potentials (eCAPs).

### 2.4. Data Analysis

Data analysis procedures were similar for both the NYU and ASAN data sets. Differences in the two data sets are noted below.

#### 2.4.1. Average CI-Aided Thresholds

For the NYU data set, the six-tone average CI-aided threshold (6-PTA) was calculated for each patient by taking the average of the 6 frequencies tested (0.25, 0.5, 1, 2, 4, and 6 kHz). Brown-Forsythe and Welch one-way ANOVA was used to compare the 6-tone average CI-aided thresholds for the Cochlear (N = 188 audiograms), AB (N = 94) and MED-EL groups (N = 34 audiograms). These tests are alternatives to the classic one-way ANOVA that do not assume homogeneity of variance or homoscedasticity, and thus are more robust. They were followed by comparisons across all three pairs of manufacturers (Cochlear/AB, AB/MED-EL, and MED-EL/Cochlear), with *p*-values adjusted for multiple comparisons. The percentage of patients in each group with a 6-PTA greater than 35 dB HL was also calculated. Fisher’s exact test was used to compare the proportion of patients with 6-PTA greater than 35 dB HL for each group. Slightly more generous than the 30 dB HL (or better) denoted in previous studies and the literature [20,21,22,23], 35 dB HL was used as the maximum upper cutoff of what we would want to achieve for CI-aided thresholds.

The same approach was used for the ASAN data set with the exceptions that instead of the 6-PTA, the five-tone average CI-aided threshold (5-PTA) was calculated for each patient by taking the average of the 5 frequencies tested (0.25, 0.5, 1, 2, and 4 kHz), and the Mann-Whitney test was used instead of ANOVA given that there were only two groups of patients (Cochlear and Med-El) rather than three.

#### 2.4.2. CI-Aided Speech Perception

For the NYU data set, CI-aided speech perception scores were separated into early and late evaluations for each manufacturer. The same methods listed above to compare 6-PTA were used to compare word scores and sentence scores between the manufacturers. This was performed separately at both the early and late timepoints.

For the ASAN data set, speech scores were analyzed using the same method we used for 5-PTA; the Mann–Whitney Rank Sum test was used. The ASAN data set only had one time point rather than two, and it used Korean rather than English words and sentences.

#### 2.4.3. Joint Analysis of CI-Aided Thresholds and Speech Perception

For the NYU data set, six-tone average CI-aided thresholds (6-PTAs) and CI-aided speech perception scores were plotted in a two-dimensional space, separately for both early and late evaluations. Joint analysis was completed separately for each evaluation time point for Cochlear, AB, and MED-EL. Pearson correlation coefficients were calculated for the 6-PTAs and all three speech perception measures: CNC words, AzBIO sentences in quiet, and AzBIO sentences in noise. For those sets of variables with a significant correlation, linear regression analysis was completed. Fisher’s r-to-z transformation was used to compare these correlations between manufacturers.

For the ASAN data set, the same analysis was completed using the five-tone average CI-aided thresholds (5-PTAs) and CI-aided word and sentence speech perception scores.

#### 2.4.4. Additional Analyses of NYU Data Set: Newer and Older

In addition to the analysis of the full NYU data set (listed above), we also separately analyzed newer (cochlear implant surgery dates between June 2018 and May 2021) and older (cochlear implant surgery dates between January 2014 and March 2016) NYU data for each manufacturer. Motivation for the comparison of newer and older data stemmed from the discussion of the introduction of AB’s Softvoice feature (introduced in 2017). Another difference deals with MED-EL patients: the newer MED-EL data has all T-levels set at 8 or 10% of the MCL level; the older MED-EL data has more variability in T-level setting (11 of 19 evaluations had T-levels set to 0, 6 of 19 evaluations had T-levels between 7 and 12% of M-levels, and 2 of 19 evaluations had measured T-levels).

## 3. Results

### 3.1. NYU Exploratory Data Set

**Figure 2:**
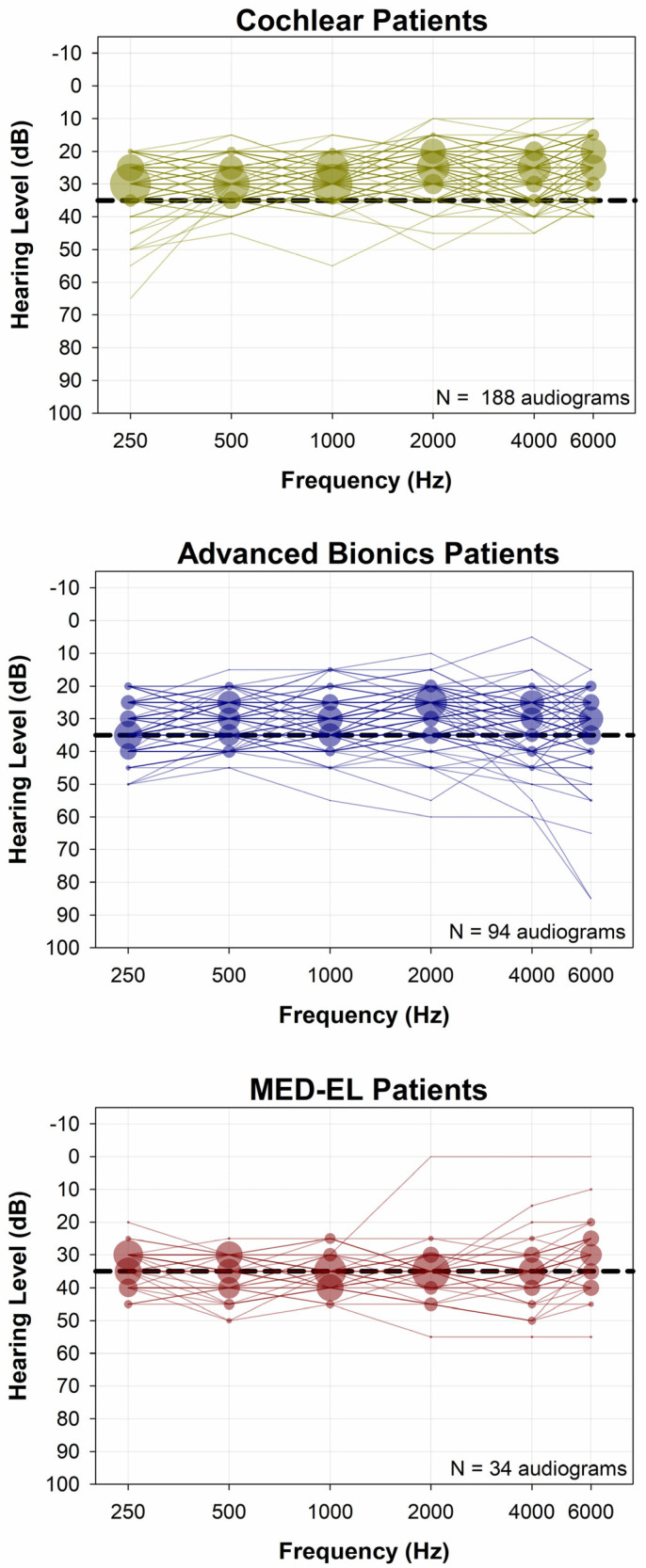
CI-aided soundfield audiograms. Symbol size represents the percentage of evaluations in each group that had a CI-aided hearing level at each respective X, Y coordinate. A dashed line at 35 dB HL is shown as a reference line. When compared to the Cochlear group, the AB and MED-EL groups had more variability in CI-aided soundfield thresholds, and more CI-aided thresholds poorer than 35 dB HL.

**Figure 3:**
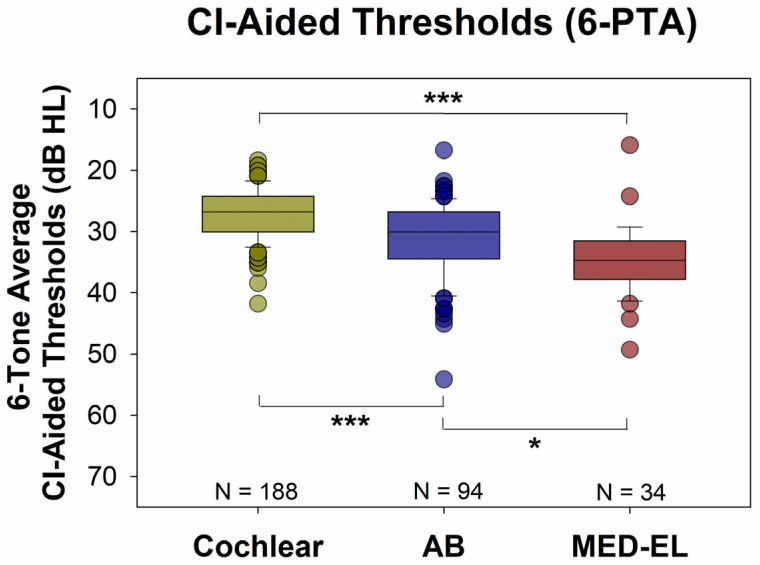
Six-tone average CI-aided thresholds (6-PTAs) for both groups. The 6-PTAs were significantly higher (poorer) for AB and MED-EL patients than for Cochlear patients (Mann-Whitney Rank Sum Test, *p* < 0.001, median: 30.0 and 34.6 dB HL vs. 26.7 DB HL). * = *p* < 0.05, *** = *p* < 0.001.

**Figure 4:**
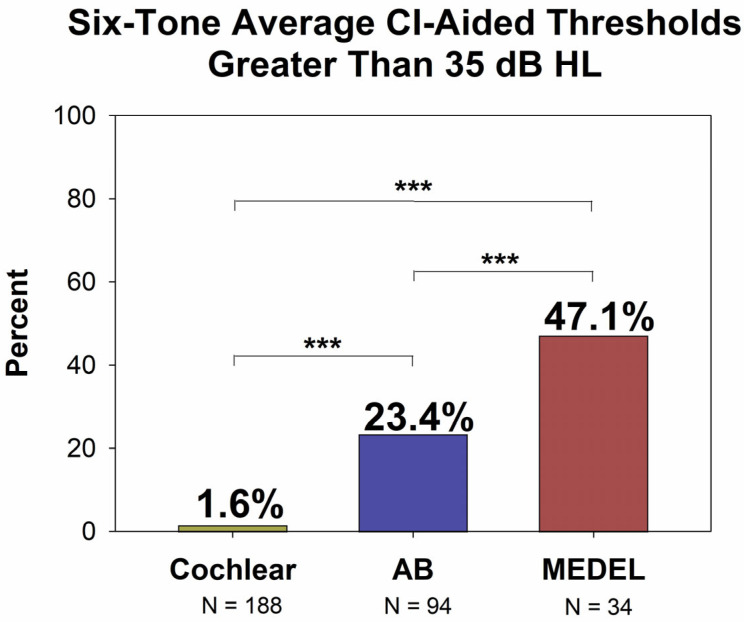
Percentage of each group with 6-PTAs above 35 dB HL. Compared to the Cochlear group, the AB and MED-EL groups had more than 10× the number of patients with a 6-PTA greater than 35 dB HL (23.4% and 47.1% vs. 1.6%). *** = *p* < 0.001.

Figure 2 shows all CI-aided audiograms for each manufacturer. Note that further down on the graph indicates a poorer hearing level. For Cochlear device users (Figure 2, top panel), the majority of thresholds were below (better than) 35 dB HL. In contrast, patients with MED-EL and AB devices (Figure 2, middle and bottom panels) CI-aided audiograms and thresholds were more likely to be above (poorer than) 35 dB HL. Figure 3 offers another way to look at the data presented in Figure 2. Figure 3 shows the distribution of the six-tone average CI-aided thresholds (6-PTAs) for each manufacturer. The 6-PTAs were significantly lower (*p* < 0.001) for Cochlear patients (mean = 27.1 dB HL, median = 26.7 dB HL) than for AB patients (mean = 31.1 dB HL, median = 30.0 dB HL) or MED-EL patients (mean = 34.6 dB HL, median = 34.6 dB HL). Post-hoc comparisons yielded adjusted *p*-values of *p* < 0.001 for Cochlear vs. AB or MED-EL, and *p* = 0.016 for AB vs. MED-EL. Additionally, MED-EL and AB device users had a significantly higher incidence of 6-PTAs greater than 35 dB HL, when compared to Cochlear device users (Fisher’s exact test, *p* < 0.001). The percentage of 6-PTAs greater than 35 dB HL was 1.6% for Cochlear users, 23.4% for AB users and 47.1% for MED-EL users (see Figure 4).

**Figure 5:**
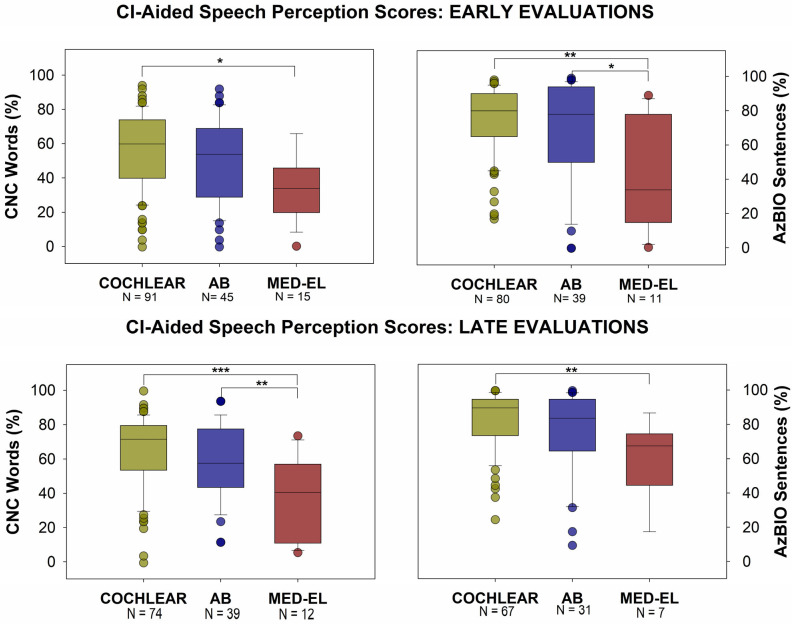
CNC word scores and AzBIO-Q sentence scores were significantly different between the groups for both early and late evaluation timepoints (One-way ANOVA, CNC: *p* = 0.0133 and *p* = 0.0003, AzBIO-Q: *p* = 0.0020 and *p* = 0.0063). At both timepoints, CNC and AzBIO-Q were higher for the Cochlear group (CNC means: 56.6% and 65.5%; AzBIO-Q means: 74.5% and 83.1%) than for the MED-EL group (CNC means: 39.1%, *p* = 0.0152 and 37.8%, *p* = 0.0002; AzBIO-Q means: 47.2%, *p* = 0.0017 and 58.9%, *p* = 0.0078). Compared to the MED-EL group, the AB group also showed significantly higher CNC scores at the late timepoint (mean: 59.38% vs. 37.83%, *p* = 0.0092) and significantly higher AzBIO-Q scores at the early timepoint (mean: 47.18%, *p* = 0.0017). * = *p* < 0.05, ** = *p* < 0.01, *** = *p* < 0.001.

When considering CI-aided speech perception scores, there were also notable differences between the groups. Figure 5 shows CI-aided speech perception scores (CNC words and AzBIO sentences in quiet) for both early and late timepoints. One-way ANOVA revealed statistically significant differences in CNC word scores among manufacturers at both early (F(2, 148) = 4.445, *p* = 0.0133) and late evaluation timepoints (F(2, 122) = 8.505, *p* = 0.0003). Post-hoc Tukey analysis indicated that CNC word scores were significantly higher for Cochlear device users (mean: 56.62% for early data and 65.54% for late data) compared to MED-EL users at the early timepoint (mean: 39.07%, *p* = 0.0152) and compared to MED-EL users at the late timepoint (mean: 37.83%, *p* = 0.0002). AB users also showed significantly higher scores than MED-EL users at the late timepoint (mean: 59.38% vs. 37.83%, *p* = 0.0092). There was no significant difference between Cochlear and AB users at either time point (early: *p* = 0.2626; late: *p* = 0.3295). The data did not violate assumptions of homogeneity of variance, as indicated by non-significant Brown-Forsythe and Bartlett’s tests (*p* > 0.05 for all comparisons).

One-way ANOVA revealed statistically significant differences in AzBio sentence scores in quiet among manufacturers at both early (F(2, 127) = 6.516, *p* = 0.0020) and late evaluation timepoints (F(2, 102) = 5.326, *p* = 0.0063). Post-hoc Tukey analysis indicated that AzBio scores were significantly higher for Cochlear device users (mean: 74.51%) compared to MED-EL users (mean: 47.18%, *p* = 0.0017) at the early timepoint. Similarly, AB users (mean: 67.72%) showed significantly higher scores than MED-EL users at the early timepoint (*p* = 0.0364). At the late timepoint, Cochlear users (mean: 83.13%) maintained significantly higher scores compared to MED-EL users (mean: 58.86%, *p* = 0.0078), while the difference between AB (mean: 76.06%) and MED-EL users was not statistically significant (*p* = 0.1028). There was no significant difference between Cochlear and AB users at either time point (early: *p* = 0.3211; late: *p* = 0.2367). Tests for homogeneity of variance showed significant differences in variance at the early timepoint (Brown-Forsythe test: *p* = 0.0334; Bartlett’s test: *p* = 0.0092), but not at the late timepoint (*p* > 0.05 for both tests). Lastly, there were no significant differences among manufacturers in AzBio sentences in noise at either early or late timepoints.

**Figure 6:**
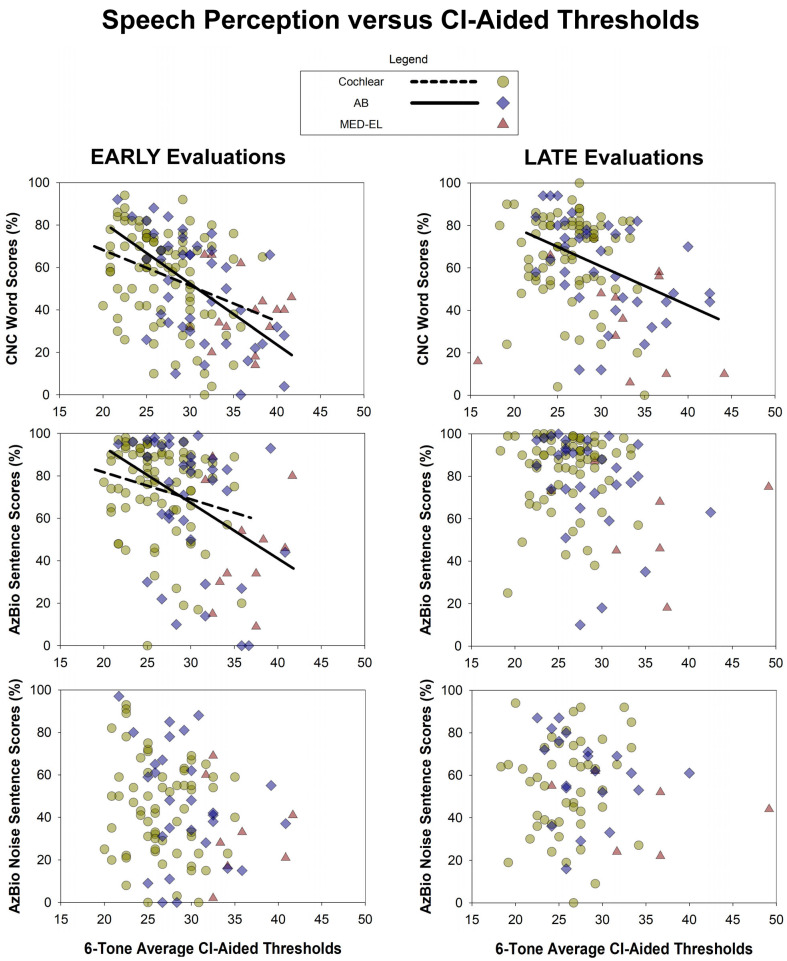
Relationship between 6-PTAs and CI-aided speech perception scores. Higher CI-aided thresholds were associated with lower speech perception scores for both early and late evaluations. Nearly all correlations were negative (15 out of 18), with five of them reaching statistical significance.

In Figure 6, we found negative correlations between 6-PTAs and nearly all speech perception conditions (15 out of 18 correlations), with five of them reaching statistical significance. See Table 1 for more information. Correlations between 6-PTAs and speech perception scores were not significantly different between manufacturers (Fisher’s r-to-z transformation, *p* > 0.05).

Comparisons of newer and older NYU data (found in Supplemental Digital Content, Figures S1–S4) did not reveal any statistically significant or meaningful differences beyond those shown in the main results.

### 3.2. ASAN Confirmatory Data Set

**Figure 7:**
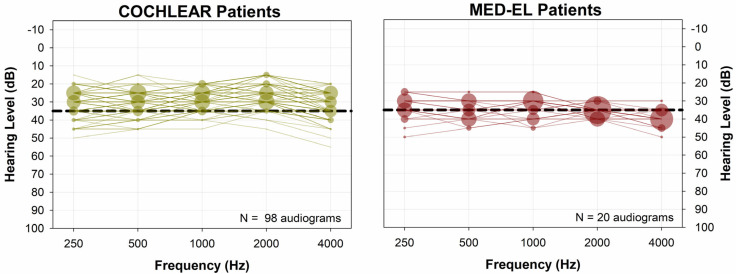
CI-aided soundfield audiograms for the ASAN dataset. Symbol size represents the percentage of the group with CI-aided hearing levels at each respective X, Y coordinate. A dashed line at 35 dB HL is shown as a reference line. When compared to the Cochlear group, the MED-EL group had more CI-aided thresholds poorer than 35 dB HL.

**Figure 8:**
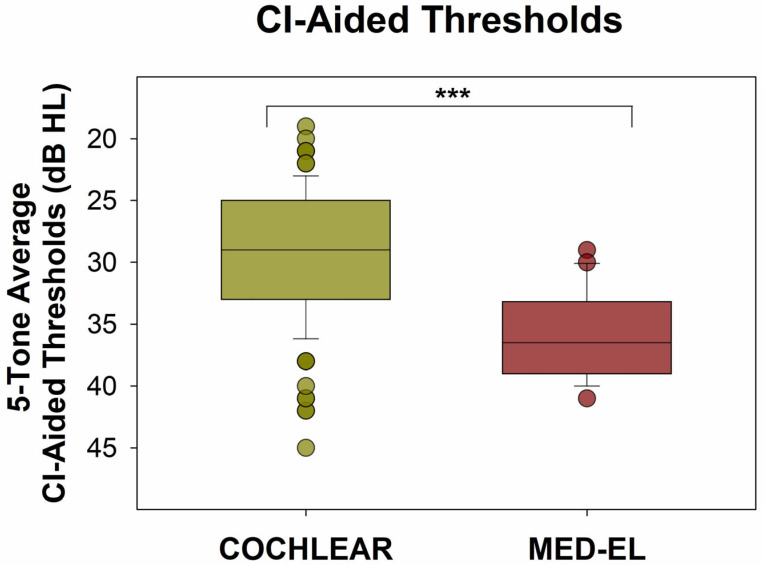
Five-tone average CI-aided thresholds (5-PTAs) for both groups. The 5-PTAs were significantly higher (poorer) for the MED-EL users than for the Cochlear users (Mann-Whitney Rank Sum Test, *p* < 0.001, median: 36.5 dB HL vs. 29.0 DB HL). *** = *p* < 0.001.

**Figure 9:**
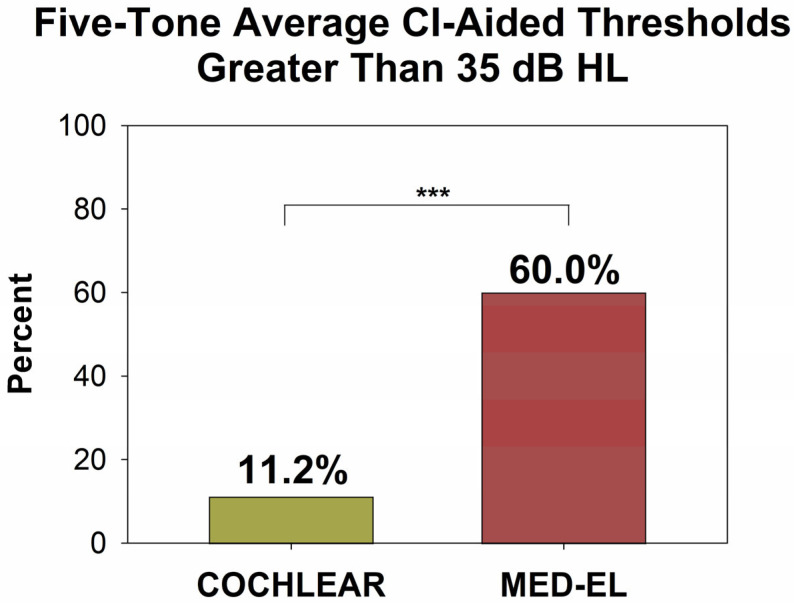
Percent of each group with 5-PTAs above 35 dB HL. Compared to the Cochlear group, the MED-EL group has nearly 5× the number of patients with 5-PTAs greater than 35 dB HL (60.0% vs. 11.2%, Fisher’s exact test, *p* < 0.001). *** = *p* < 0.001.

Results from our confirmatory data set are presented in the same way as those from the NYU data set. Figure 7 shows all CI-aided audiograms for the data set provided by the ASAN Clinic. Similar to the NYU data set, Cochlear device users (Figure 7, left panel) of our confirmatory data set had most CI-aided thresholds falling below (better than) 35 dB HL. In contrast, patients with MED-EL devices exhibit higher thresholds, more frequently exceeding (poorer than) 35 dB HL. This descriptive analysis is further confirmed by the information presented in Figure 8 and Figure 9. Figure 8 illustrates the distribution of five-tone average CI-aided thresholds (5-PTAs) for each group. Compared to Cochlear users, 5-PTAs were significantly higher (poorer) for MED-EL users (median: 29 dB HL vs. 36.5 dB HL; Mann-Whitney Rank Sum Test, *p* < 0.001). Additionally, Figure 9 shows that MED-EL users had a significantly higher incidence of 5-PTAs greater than 35 dB HL, when compared to Cochlear users (60.0% vs. 11.2%; Fisher’s exact test, *p* < 0.001).

**Figure 10:**
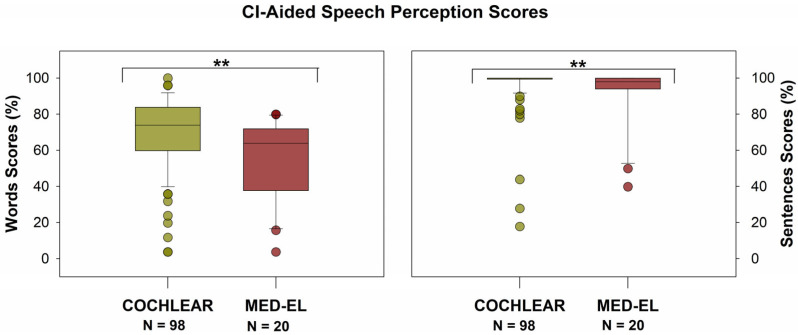
Speech perception scores were significantly higher for Cochlear users than for MED-EL users for both words (median: 74% vs. 64%, mean: 69.6% vs. 55.9%; Mann–Whitney Rank Sum test, *p* < 0.01) and sentences (median: 100% vs. 98%, mean: 96.4% vs. 91.5%; Mann–Whitney Rank Sum test, *p* = 0.01). ** = *p* < 0.01.

**Figure 11:**
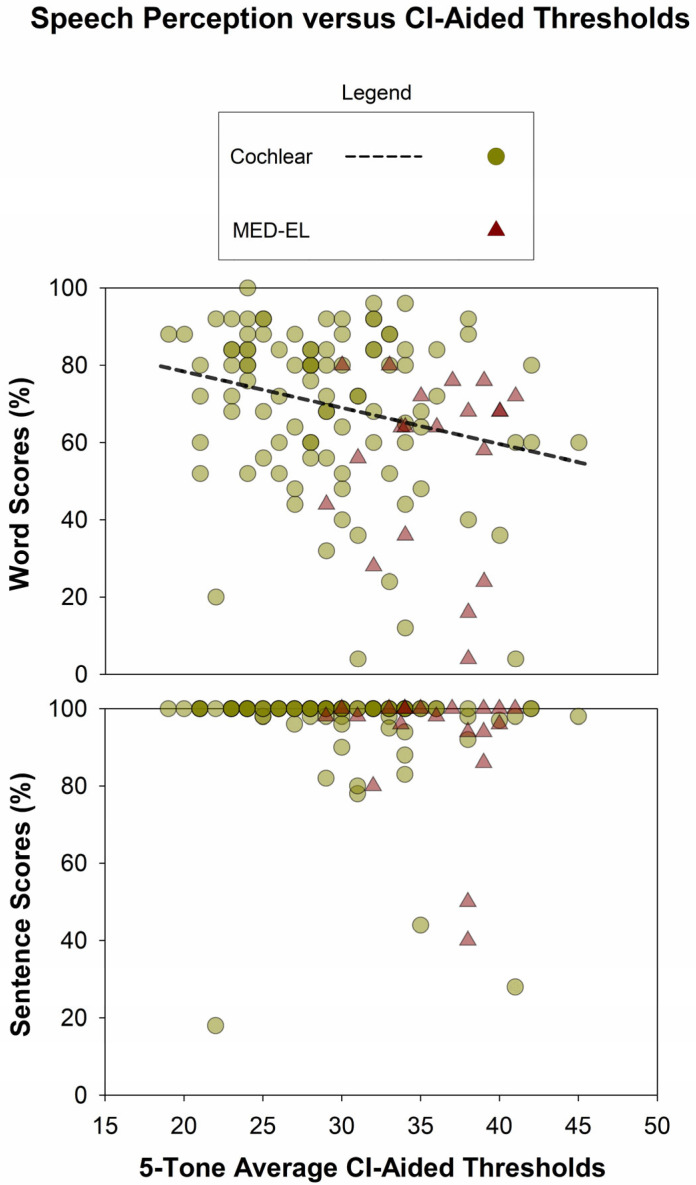
Relationship between 5-PTAs and CI-aided speech perception scores. All correlations were negative, with one of those reaching statistical significance.

CI-aided speech perception scores were significantly higher for Cochlear users than for MED-EL users for word scores (median: 74% vs. 64%, mean: 69.6% vs. 55.9%; Mann–Whitney Rank Sum test, *p* < 0.01) and sentence scores (median: 100% vs. 98%, mean: 96.4% vs. 91.5%; Mann–Whitney Rank Sum test, *p* = 0.01), as shown in Figure 10. Notably, the sentence scores show a pronounced ceiling effect. The relationship between 5-PTAs and CI-aided speech perception scores is shown in Figure 11: All correlations were negative, and one of those was statistically significant. See Table 2 for additional information.

## 4. Discussion

This retrospective study compared CI-aided thresholds and speech perception across different cochlear implant manufacturers. Most Cochlear users had average CI-aided thresholds better than 35 dB HL, while many AB and MED-EL users showed reduced audibility (5-PTA > 35 dB HL or 6-PTA > 35 dB HL). Our results suggest aided soundfield audiograms are especially important for patients with estimated rather than behaviorally-measured lower stimulation levels. This provides a target for intervention: patients whose CI-aided thresholds are worse than a particular level (35 dB HL was selected as a reasonable value in this study) may benefit from reprogramming approaches that differ from the standard manufacturer recommendations.

Notably, we found a weak but significant correlation between CI-aided speech perception scores and CI-aided thresholds, suggesting that higher thresholds may be associated with lower speech perception. This aligns with Chang et al. [20], who demonstrated improved speech recognition when MED-EL programming was optimized for aided thresholds between 25 and 35 dB HL (compared to 15–25 dB HL or 35–45 dB HL).

We utilized exploratory (NYU) and confirmatory (ASAN) datasets with pre-registered hypotheses and analysis methods. Two of the pre-registered hypotheses were validated: MED-EL users had a greater incidence of PTA > 35 dB HL, and correlations between word recognition scores and PTA were negative. The third hypothesis—that this correlation would be stronger for MED-EL than Cochlear users—was not supported.

A limitation of the current retrospective study is the confound between programming approaches (primarily how T-levels are determined) and the manufacturer. Cochlear patients had measured T-levels, while most AB and MED-EL patients had estimated T-levels. Another limitation is the difference in group sizes, specifically the small number of MED-EL patients in the NYU data set. Based on the present findings, we hypothesize that the practice of estimating T-levels may contribute to reduced audibility and potentially reduced speech perception scores in some patients.

The reader might be tempted to draw conclusions about speech perception with different devices, but it is important to emphasize that this study was not designed to compare outcomes among manufacturers, despite what the data might suggest. There are several methodological reasons for this. First, patients were not randomly assigned to device manufacturers, creating potential selection bias through unmeasured variables that could influence outcomes. Second, as shown before, poorer PTA thresholds correlate with worse speech perception outcomes, with these poorer thresholds predominantly concentrated in manufacturers that recommend estimated T-levels. Therefore, it is reasonable to hypothesize that implementing measured T-levels (particularly in patients with PTA > 35 dB HL) would likely improve outcomes for this subset of patients, potentially reducing or eliminating any apparent performance differences across manufacturers.

Previous small-scale studies found no significant differences in CI-aided thresholds or speech perception between measured versus estimated T-levels. Boyd [24] (N = 8/12) showed non-significant 5 dB improvements with behavioral T-levels in MED-EL users, though the proportion of subjects with PTA > 35 dB HL dropped from 25% to 0%. Similarly, Spahr & Dorman [25] (N = 15) found no significant speech perception differences across three T-level programming methods. However, these studies were small (12 to 15 subjects), and while they did fail to find a statistically significant difference, they did not claim equivalence of outcomes (see Fleming [26] for a description of equivalence and noninferiority trials). Thus, the analysis of potential differences in outcomes when using measured vs. estimated T-levels remains an open question. Moreover, related research by Dawson et al. [27] and Busby & Arora [28] observed decreased speech perception when T-levels were systematically lowered below psychophysical thresholds. Along the same lines, Martins et al. [29] (N = 30, Cochlear Nucleus system), found that when T-levels were lowered by 10 clinical units from the behaviorally measured electrical thresholds, CI-aided thresholds were 1 to 2 dB poorer than results obtained with the behavioral map. This makes sense- if we decrease T-levels below the behavioral thresholds, the corresponding T-SPL will be inaudible, thus needing louder acoustic stimulation to reach an audible electrical stimulation level.

Our findings suggest that patients who are programmed with estimated T-levels, whose CI-aided audiograms show reduced audibility, might benefit from adjusting T-levels closer to the actual psychophysical thresholds. An important benefit of estimating instead of measuring T-levels is that it reduces the amount of time it takes to program a processor. However, taking the time to measure even a subset of T-levels across the array may ultimately save the additional time requirement of repeating an aided audiological evaluation. While estimated T-levels may be a very reasonable starting point, for a substantial portion of patients (those with reduced audibility), further T-level adjustments may be warranted. Future research directions will focus on prospective data collection, by comparing the effect of measured vs. estimated T-levels within-subject (and thus, within-manufacturer), thus providing a stronger foundation for our present recommendations.

## Figures and Tables

**Table 1:** Correlation information for 6-PTAs and CI-aided speech perception scores for (A) early evaluations and (B) late evaluations. * = *p* < 0.05, ** = *p* < 0.01, *** = *p* < 0.001.

A. EARLY EVALUATIONS								
CNC Words			AzBIO Sentences in Quiet			AzBIO Sentences in Noise		
COCHLEAR	AB	MED-EL	COCHLEAR	AB	MED-EL	COCHLEAR	AB	MED-EL
r = −0.318	r = −0.559	r = −0.135	r = −0.221	r = −0.363	r = 0.013	r = −0.145	r = −0.274	r = −0.165
** *p* = 0.002	*** *p* < 0.001	*p* = 0.632	* *p* = 0.047	* *p* = 0.027	*p* = 0.971	*p* = 0.252	*p* = 0.158	*p* = 0.697
N = 91	N = 43	N = 15	N = 81	N = 37	N = 11	N = 64	N = 28	N = 8
Slope = 1.67	Slope = 2.88	--	Slope = 1.27	Slope = 2.62	--	--	--	--
B. LATE EVALUATIONS								
CNC Words			AzBIO Sentences in Quiet			AzBIO Sentences in Noise		
COCHLEAR	AB	MED-EL	COCHLEAR	AB	MED-EL	COCHLEAR	AB	MED-EL
r = −0.170	r = −0.448	r = −0.187	r = 0.059	r = −0.298	r = −0.134	r = 0.110	r = −0.192	r = −0.256
*p* = 0.148	** *p* = 0.005	*p* = 0.561	*p* = 0.633	*p* = 0.110	*p* = 0.774	*p* = 0.461	*p* = 0.418	*p* = 0.624
N = 74	N = 38	N = 12	N = 67	N = 30	N = 7	N = 47	N = 20	N = 6
--	Slope = 1.84	--	--	--	--	--	--	--

**Table 2:** Correlation information for 5-PTAs and CI-aided speech perception scores. * = *p* < 0.05.

ASAN EVALUATIONS			
Words		Sentences	
COCHLEAR	MED-EL	COCHLEAR	MED-EL
r = −0.246	r = −0.0157	r = −0.144	r = −0.194
* *p* = 0.0145	*p* = 0.948	*p* = 0.157	*p* = 0.413
N = 98	N = 20	N = 98	N = 20
Slope = 0.937	--	--	--

## Data Availability

The raw data supporting the conclusions of this article will be made available by the authors on request.

## Supplementary Materials

The following supporting information can be downloaded at: https://www.mdpi.com/article/10.3390/audiolres15040079/s1, Figure S1: 6-Tone Average CI-Aided Thresholds; Figure S2: CI-Aided Thresholds Greater Than 35 dB HL; Figure S3: CI-Aided Speech Perception Scores; Figure S4: Speech Perception versus CI-Aided Thresholds.
