# Supplementary material for: Cochlear Implants and the Aided Audiogram: A Retrospective Study Comparing Performance Across Device Manufacturers
Source: Audiol Res. 2025 Jul 2;15(4):79. doi: 10.3390/audiolres15040079 (PMC12286021; doi:10.3390/audiolres15040079)
Supplement: Supplementary file 1 [file audiolres-15-00079-s001.zip › Figure S4.pdf]

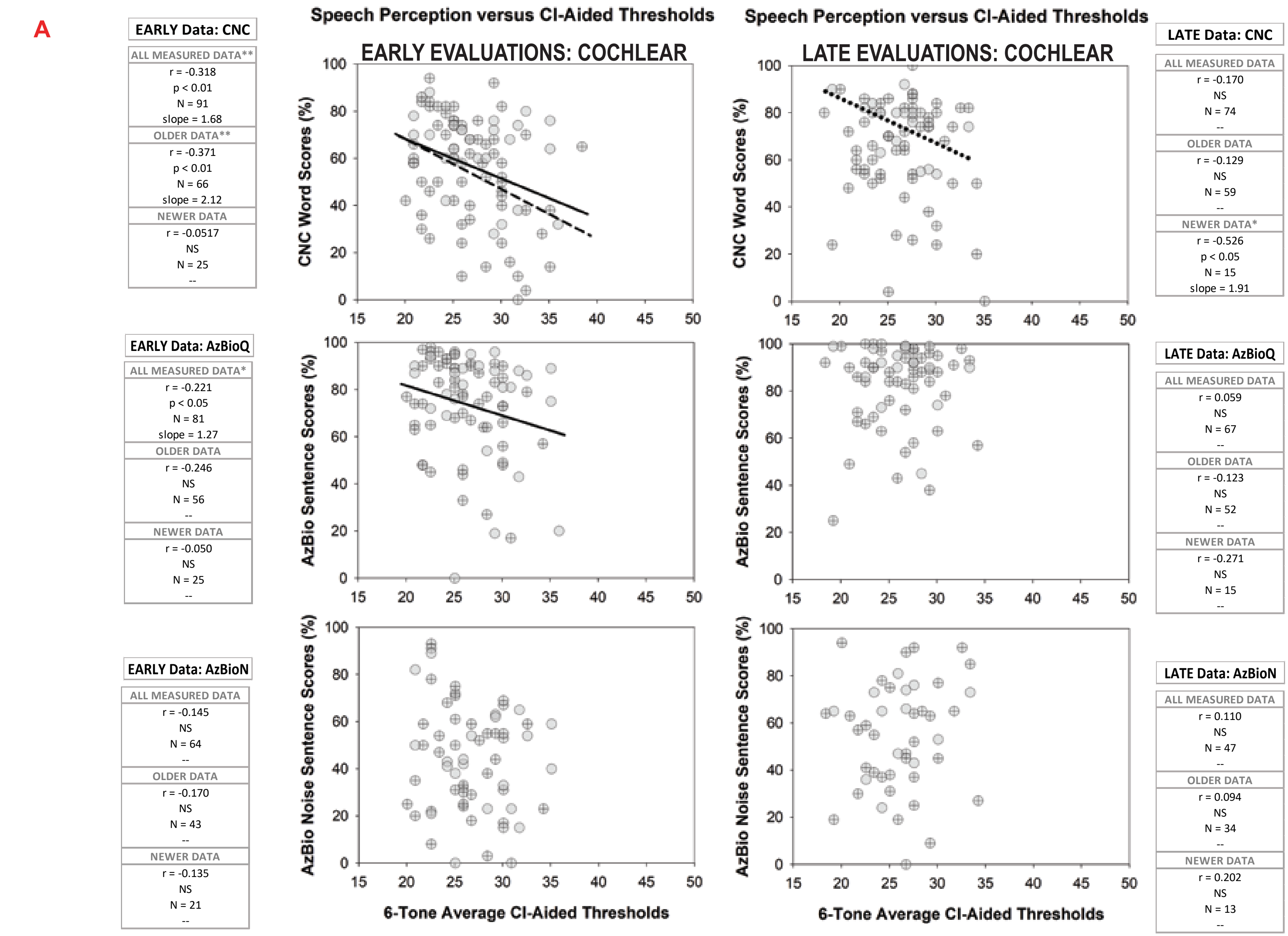

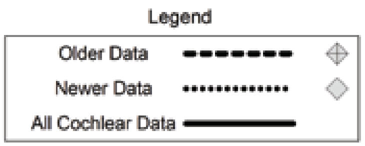

CI-Aided Speech Perception versus CI-Aided Thresholds. Panel A is Cochlear data comparison of *newer* (cochlear implant surgery dates between June 2018 and May 2021) and *older* (cochlear implant surgery dates between January 2014 and March 2016) data. N is the number of evaluations.

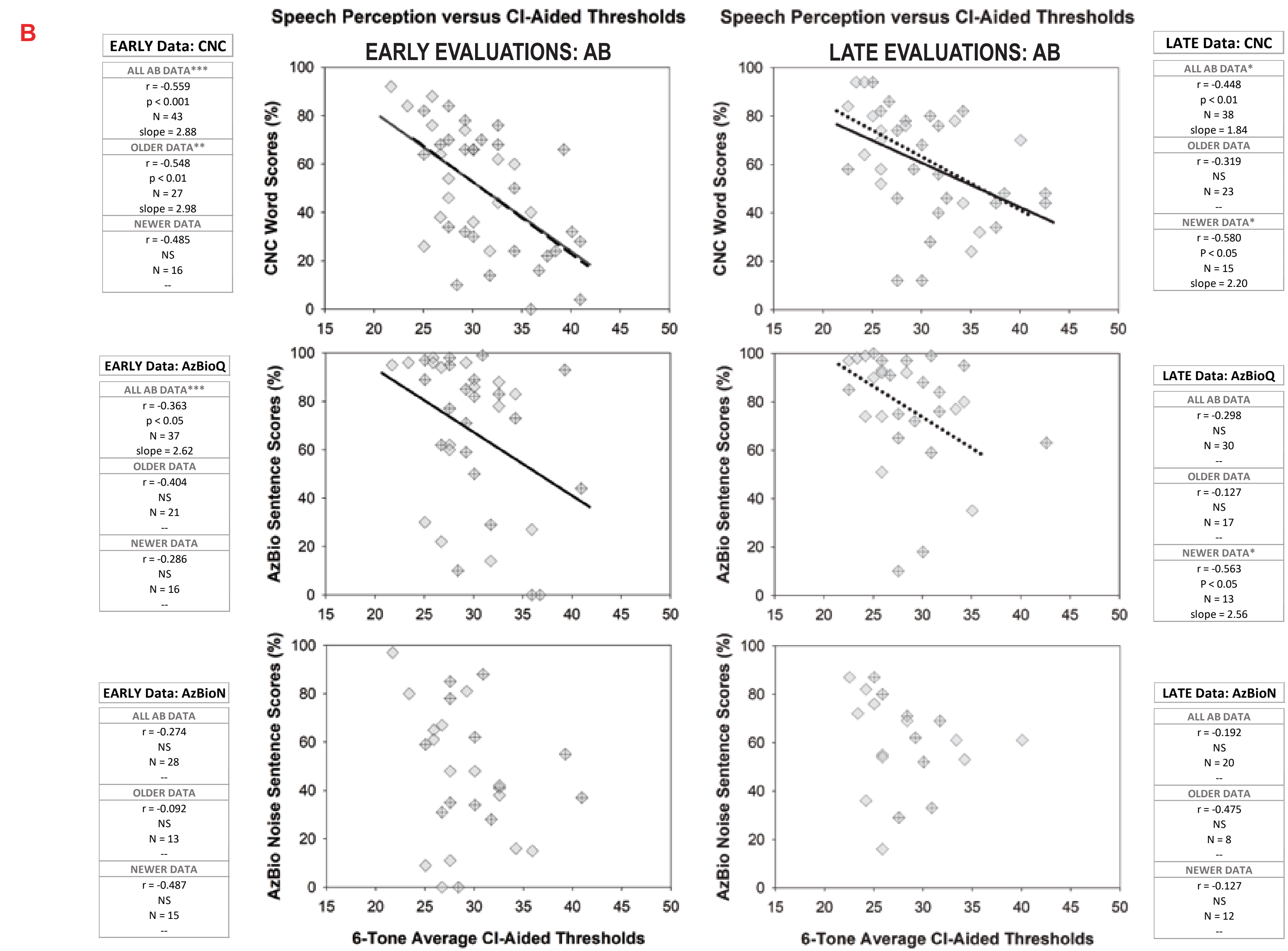

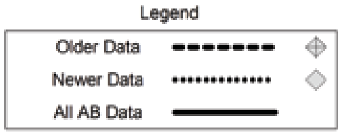

CI-Aided Speech Perception versus CI-Aided Thresholds. Panel B is the AB data comparison of *newer* (cochlear implant surgery dates between June 2018 and May 2021) and *older* (cochlear implant surgery dates between January 2014 and March 2016) data. N is the number of evaluations.

C

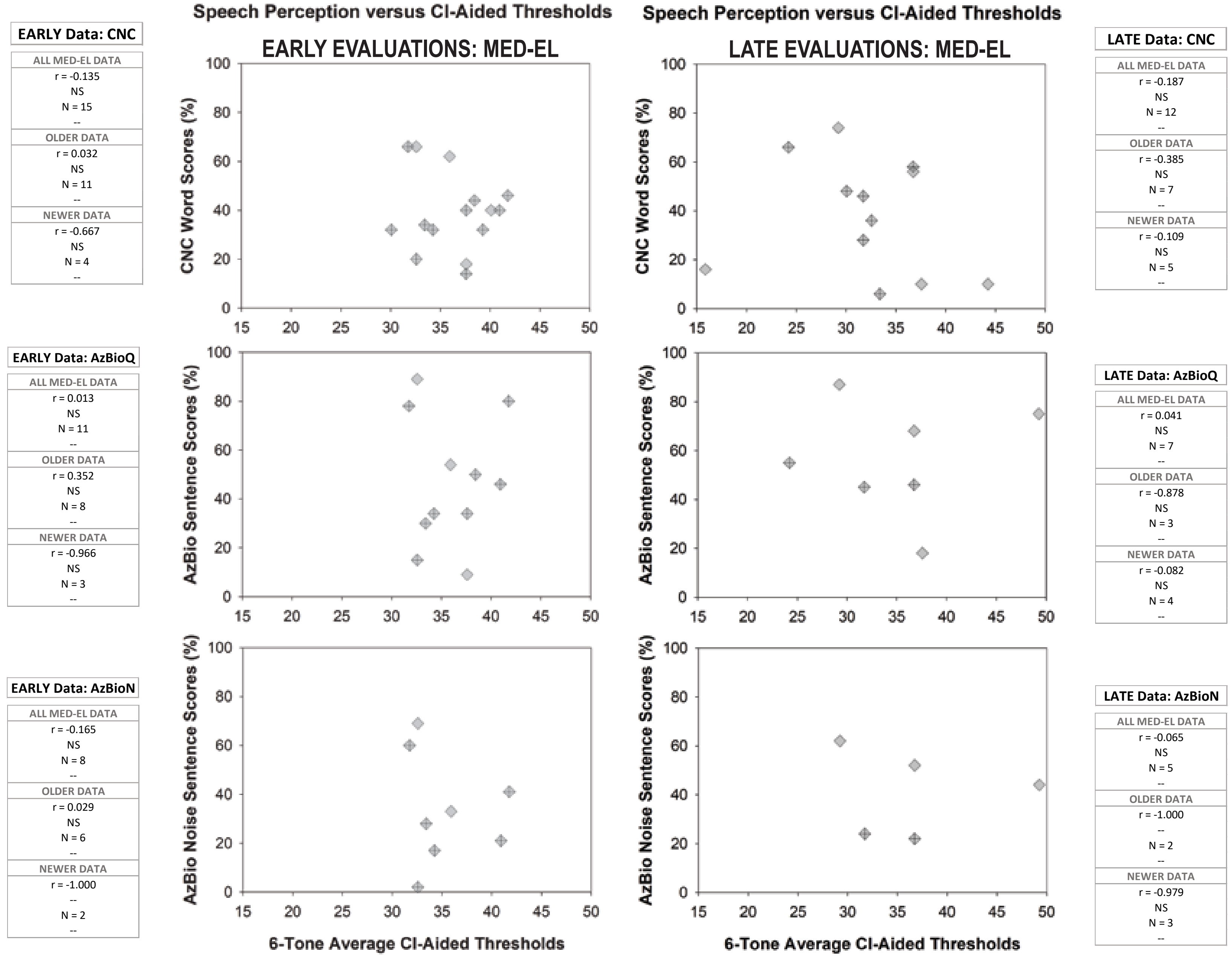

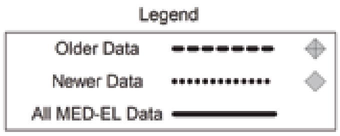

CI-Aided Speech Perception versus CI-Aided Thresholds. Panel C is the MED-EL data comparison of *newer* (cochlear implant surgery dates between June 2018 and May 2021) and *older* (cochlear implant surgery dates between January 2014 and March 2016) data. N is the number of evaluations.
